# *Opuntia* spp. Fibre Characterisation to Obtain Sustainable Materials in the Composites Field

**DOI:** 10.3390/polym13132085

**Published:** 2021-06-24

**Authors:** Jessica Castellano, María D. Marrero, Zaida Ortega, Francisco Romero, Antonio N. Benitez, Myriam R. Ventura

**Affiliations:** 1 Departamento de Ingeniería Mecánica, Universidad de Las Palmas de Gran Canaria, 35017 Las Palmas, Spain; mariadolores.marrero@ulpgc.es (M.D.M.); francisco.romero@ulpgc.es (F.R.); 2 Departamento de Ingeniería de Procesos, Universidad de Las Palmas de Gran Canaria, 35017 Las Palmas, Spain; zaida.ortega@ulpgc.es (Z.O.); antonionizardo.benitez@ulpgc.es (A.N.B.); 3 Departamento de Patología Animal, Producción Animal, Bromatología y Tecnología de los Alimentos, Universidad de Las Palmas de Gran Canaria, 35413 Las Palmas, Spain; myriam.rodriguezventura@ulpgc.es

**Keywords:** *Opuntia*, vegetal fibre, fibre treatment, chemical composition, FTIR

## Abstract

Some studies have evaluated the use of *Opuntia* as reinforcement for polymeric matrices, obtaining good results in energy absorption tests and increasing the tensile elastic modulus. However, no studies focusing on the previous characterisation of the fibres and their treatment to improve compatibility with polymeric matrices have been found. This work analyses the chemical composition of *Opuntia maxima* (OM) and *Opuntia dillenii* (OD) cladodes and fibre, studying how different treatments influence it. AOAC 2000 methods were used to determine non-structural components and the Van Soest method was used to estimate structural components. Surface characteristics of the samples were also evaluated by Fourier Transform Infrared Spectroscopy (FTIR). *Opuntia* fibre presented higher cellulose (50–66%) and lignin (6–14%) content and lower hemicellulose (8–13%) content than *Opuntia* cladodes (9–14% cellulose, 20–50% hemicellulose, 1–4% lignin). Despite the variability of lignocellulosic materials, OD cladodes treated with water and acetic acid achieved an increase in the structural components. Alkaline fibre treatment removed pectin and hemicellulose from the fibre surface, slightly increasing the cellulose content. Future research should evaluate whether the treated *Opuntia* fibre can improve the mechanical properties of reinforced polymer.

## 1. Introduction

Composites with vegetal fibres have received a lot of attention due to the need to develop materials that have new properties and that are more respectful to the environment at the same time. During the last years, vegetal fibre demand has increased in the industrial sector, where its use is a trend thanks to its low density, low cost, low energy consumption, and biodegradability [1,2], among other factors, compared to synthetic fibres. However, they have some disadvantages, such as variable properties, matrix incompatibility, and moisture sensitivity [3,4,5,6]. Since the 1990s, vegetal-fibre-reinforced polymer composites have been used as an alternative to synthetic fibre reinforcements in different applications, mainly in the automotive [7,8] and construction sectors [9,10,11]. Nevertheless, new vegetal fibres with lower cost and greater availability than the commonly studied and used ones need to be evaluated [12].

In this sense, *Opuntia*, also known as barbary fig, cactus pear, and prickly pear [13], is presented as a candidate to consider. It is a species belonging to the *Cactaceae* family that has historically been used as food [14]. Its main body is formed by articulated stems called cladodes, pads, or “*nopalitos*” (the youngest) with a flattened padded growth form [15]. Spines and mucilage are characteristic elements of this plant [16]. This genus has an asynchronous reproduction and Crassulacean Acid Metabolism (CAM), a photosynthetic adaptation to environmental stress that allows it to grow with a high level of efficiency under limited water conditions, adapting to arid areas and adverse environments easily [17].

Inside the *Opuntia* cladodes, there is a fibre network similar to a skeleton, with the same shape as the pad that it supports. Different processes have been evaluated to extract this fibre network, such as water retting [18] or burial [19,20] methods. On the other hand, this skeleton can be collected directly from nature: when cladodes finish their life cycle they tend to dry out and lose part of their greenish elements, leaving only the fibre network structure, which can be directly exploited [21,22].

*Opuntia* is considered an invasive species in different parts of the world, so frequent pruning is required to contain its rapid growth and expansion [23]. Moreover, during fruit processing large amounts of waste and by-products are generated [24,25]. Consequently, a lot of waste is obtained that can be used and evaluated as reinforcement of composites.

Mannai et al., (2018) have valued the *Opuntia* fibre network extraction, and also its structure and properties, showing good mechanical responses [18]. Other studies have analysed the use of the *Opuntia* ground cladodes directly as reinforcement [26,27], and also the *Opuntia* fibre [28,29]. Matrices such as polylactic acid [21,28,30], polypropylene [26,31], high density polyethylene [27], and polyester [19] have been reinforced with *Opuntia*, mainly by compression moulding, offering good results in energy absorption tests and increasing the tensile elastic modulus (for example, from 800 MPa for net polyester to 1480 MPa for the composite [19]). However, no studies focusing on the previous chemical characterisation of the fibres and their treatment to improve compatibility with polymeric matrices have been found. Chemical treatments can improve the interfacial adhesion and enhance the composite mechanical properties [32,33].

The research to date has tended to focus on *Opuntia* cladodes composition rather than *Opuntia* fibre composition. It is due to the multiple uses of *Opuntia* pads, which have been evaluated, such as raw materials for bioethanol production [17] or nutritional supplements, showing functional properties like antidiabetic, antihyperglycemic, and hypoglycemic effects [34,35]. Moreover, the treatment of cladodes with water, ethanol, and lemon juice [36] or with NaOH and KOH [37] have been reported, but information about *Opuntia* fibre treatment is not found in the literature. 

Considering that vegetal fibres’ composition affects their properties as reinforcement [7], a chemical characterisation of the *Opuntia* fibre is needed to enhance its use as reinforcement of polymeric matrices. This constitutes the main novelty of this study due to the lack of information on the issue. Vegetal fibres are composed of structural components (cellulose, hemicellulose, and lignin) and non-structural components (pectin, fats, waxes, etc.). This composition depends on various factors, such as time of harvesting, climatic history, soil characteristics, vegetative state or plant age, fibre extraction process, and fibre characterisation method [38,39,40]. Considering this variability, the aim of this study is to determine the chemical composition of *Opuntia* cladodes, on the one hand, and *Opuntia* fibre, on the other, analysing how different treatments influence them.

## 2. Materials and Methods

### 2.1. Plant Samples and Fibre Obtaining

There are two species of *Opuntia* considered invasive in the Canary Islands according to the Spanish Catalogue of Invasive Alien Species: *Opuntia maxima* (OM) and *Opuntia dillenii* (OD). OM plants can reach up to 6 m tall, and their pads may have thorns (thin and whitish) or not. On the other hand, OD plants have many ramifications, they do not exceed 3 m in height, and their pads have more thorns (thick and yellowish).

In this study, samples of 8 different wild plants (4 OM plants and 4 OD plants) have been collected in the island of Gran Canaria (Spain), using the letters A, B, C, and D to differentiate one from the other; each plant will be referred in this paper with the abbreviation of the specific species (OM or OD) and the specimen at the end (for example, OM.A refers to specimen A in *O. maxima*).

**Figure 1:**
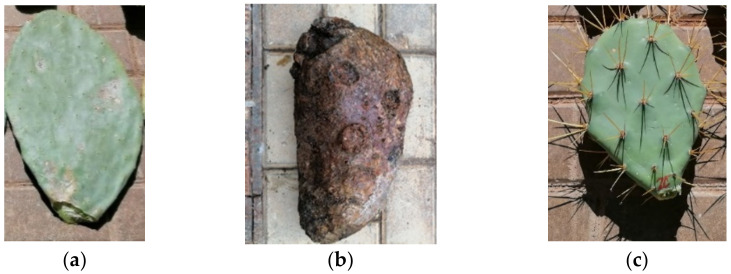
Examples of the cladodes use for the study: (**a**) OM young cladode, (**b**) OM old cladode, (**c**) OD young cladode.

**Figure 2:**
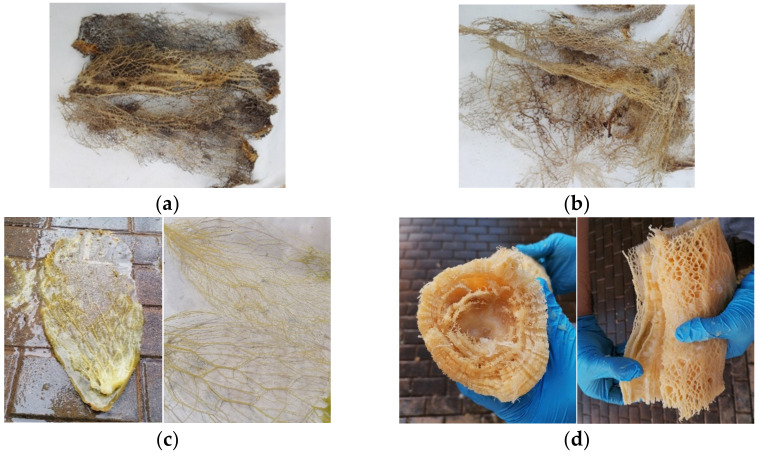
Fibre from *Opuntia*: (**a**) directly obtained from nature OM cladodes, (**b**) directly obtained from nature OD cladodes, (**c**) obtained by water retting process from young OM cladodes, (**d**) obtained by water retting process from old OM cladodes.

From plants A, B, and C of both species, three young green cladodes were collected (18 young green cladodes in total). Only three old brown cladodes from plants A, B, and C of OM were collected. Old cladodes were not collected from OD plants because of the difficulty of accessing them. Figure 1 shows the different types of pads collected. The thorns were manually removed from the cladodes for easy handling. D plants were used to obtain *Opuntia* fibre directly from nature (Figure 2a,b). In Table 1, the different samples obtained are shown.

To obtain *Opuntia* fibres under controlled conditions (apart from the fibre obtained from D plants), young and old cladodes from OM.A, OM.B, and OM.C plants were collected and immersed in water for 15–40 days in a closed container. After this time, which depended on the size of the cladode and its maturity, it was possible to obtain the fibre network manually from the interior of the cladodes. In the case of mature cladodes, it is formed by several layers, as can be seen in Figure 2d.

Considering that OD cladodes are smaller than OM, that their thorns make processing difficult, and that their old cladodes are inaccessible, OD fibres were not obtained by the water extraction process.

As shown in Figure 2a,b, fibres collected directly from nature (D plants) seem more deteriorated than those obtained after immersion in water, whose whitish colour suggests that they are in a better state.

### 2.2. Cladode Treatments

Young cladodes collected from both species were cut into cubes and subjected to the following treatments with the aim of removing part of the non-structural components:

- Stirring with water at room temperature for 2 h, avoiding the use of chemical agents and trying to simplify the process;
- Stirring with acetic acid (10% vol) for 2 h, considering that the acidic medium facilitates the separation of the fibres and the removal of part of the lignin present in the fibre surface.

After the treatments, samples were washed, filtered, dried (at room temperature and then at 60 °C in an oven) and finally ground (Retsch Ultra Centrifugal Mill ZM 200, Verder Scientific, Haan, Germany). Table 1 shows the different treated cladodes samples obtained.

The cladode treatments’ yields were calculated as the ratio of the weight of the dry cladodes’ cubes after the treatments and the weight of the fresh cladodes.

### 2.3. Fibre Treatments

It is well known that a good matrix–reinforcement interface bond is required to achieve an optimal reinforcement [41]. In this way, chemical treatment is one of the best ways to increase fibre–matrix compatibility [1]. Alkaline treatment with NaOH is a very common method in the literature: it removes the amorphous content, hemicellulose and lignin, which leads to the fibre surface becoming rough [42]. Moreover, it reduces the fibre’s hydrophilic character and water absorption [43]. On the other hand, sodium chlorite bleaching can remove lignin, and this delignification can provide an increase in the reinforcing power of the fibres, increasing the cellulose content and favouring the transfer of stress [44].

Ground fibre samples (with a size smaller than 500 µm) obtained by the water extraction process from old cladodes were subjected to the following treatments during 1 h (1:25 ratio):

- Stirring with NaOH 1 M at room temperature and at 70 °C;
- Stirring with 1% wt sodium chlorite at room temperature and at 70 °C (adjusting the pH to 4.5 with acetic acid);
- Stirring with 1% wt sodium chlorite at 70 °C (adjusting the pH to 4.5 with acetic acid) for 1 h, filtered and washed with water, and then re-immersed in a NaOH 1M solution at room temperature (for 1 h also).

These treatments have focused on fibre obtained from old cladodes because they show a higher fibre content than the young cladodes [37]. A maximum yield of 9% (dry weight) was obtained for young cladodes fibre extraction, while from old cladodes, a maximum yield of 34% (dry weight) was achieved. These results are consistent with those reported by Mannai et al., (2018): yields for young cladodes (1.6%) are lower than for old cladodes (30%) [18].

Fibre samples obtained directly from nature were only subjected to NaOH 1M treatment at room temperature.

After the treatments, samples were washed, filtered, and dried (at room temperature and then at 60 °C in an oven).

The fibre treatments yields were calculated as the ratio of the weight of the dry ground fibres after the treatments and the weight of the dry ground fibres before the treatment.

Identification of all samples obtained (46 treated and untreated samples) is summarised in Table 1.

### 2.4. Plant and Fibre Characterisation

**Figure 3:**
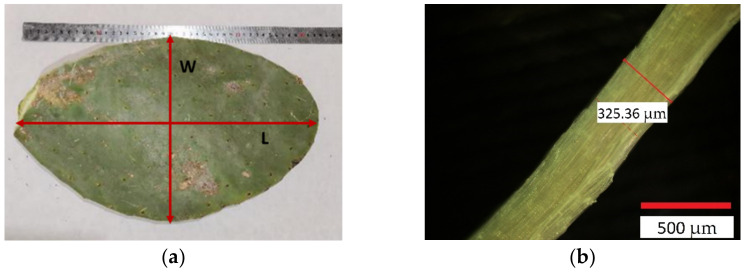
(**a**) Biometric parameters measured in the OM cladodes. L: length; W: width. (**b**) Fibre bundle width measured by an optical microscope.

The biometric parameters (length and width) of 30 cladodes from OM were measured using a ruler (Figure 3a). The width of the fibre’s bundles obtained by the water retting process was measured using an optical microscope (Olympus BX51), as can be seen in Figure 3b. A total of 600 measurements were made. The *Opuntia* fibre bundle structure was also inspected using scanning electron microscopy (SEM, Hitachi TM3030). 

Moisture, ash, and crude protein (CP) were determined according to standard methods as described in the AOAC (2000) (methods 930.15, 942.05, and 976.05, respectively).

An extraction method using a Soxhlet apparatus (with petroleum ether) during 6 h was performed to determine the ethereal extract (fats, vegetable pigments, waxes, etc.).

The structural components were estimated according to neutral detergent fibre (NDF), acid detergent fibre (ADF), and acid detergent lignin (ADL), known as Van Soest methods [45], using an ANKOM 220 Fibre Analyzer apparatus (ANKOM Technology, Macedon, New York, United States). Hemicellulose was calculated according to Equation (1), and cellulose in accordance with Equation (2):

Hemicellulose = NDF − ADF(1)

Cellulose = ADF − ADL(2)

Lignin content is equivalent to ADL content.

Surface characteristics of the samples were also analysed by Fourier Transform Infrared Spectroscopy (FTIR). FTIR spectra were obtained in a Perkin Elmer Spectrum Two apparatus (PerkinElmer, Waltham, Massachusetts, United States), equipped with an attenuated total reflectance (ATR) device, from 4000 to 500 cm^−1^, at a resolution of 4 cm^−1^, obtaining each spectrum as the average of 50 scans.

## 3. Results

### 3.1. Plant Characterisation

**Figure 4:**
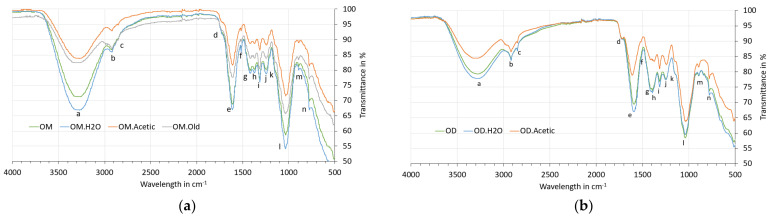
FTIR spectra for untreated and treated: (**a**) OM cladodes, (**b**) OD cladodes.

This section provides the data obtained in the cladodes’ characterisation. First, biometric parameters are presented (Table 2), then the cladodes’ composition (Table 3), and finally, the FTIR spectrum are analysed (Figure 4).

#### 3.1.1. Morphology

Biometric parameters of OM young cladodes are shown in Table 2. The collected pads have an average size of 35 cm long and 17 cm wide. These dimensions are directly related to the availability of water and nutrients. According to Pérez-Sanchez et al., the length of the blades increases in the dry season, while the width increases in the rainy season [46].

#### 3.1.2. Chemical Composition

Non-structural components of OM and OD cladodes are presented in Table 3. It can be observed that the moisture content is higher in old cladodes than in young ones, and it decreases in all pads treated with acetic acid. These results are interesting, since the acetic acid treatment can facilitate the fibre–matrix bonding if it is considered that polymeric matrices are hydrophobic.

The young pads analysed have a high ash content (18.19–20.55%). These results are in agreement with those of other studies that said that ashes are mainly calcium, potassium, and magnesium [47,48,49]. Ash content decreases in old OM cladodes (maybe because of their being less exposed to environmental factors) and in OD. Acetic samples (possibly because the acetic acid remove part of them).

Regarding CP, untreated cladodes show a content between 1.59 and 3.82%, being less in the old OM cladodes. This result may be explained by the fact that CP increases in young pads due to increased metabolic activity, and decreases in mature pads, possibly as a result of nitrogen transport from mature cladodes to young [35]. CP content increases in all samples treated with acetic acid.

The ethereal extract content is not shown in Table 3 because all samples presented a value lower than 2.5% (2.09% wt on average).

Table 3 also provides the experimental data on structural components of cladodes. For OM cladodes, it can be observed that the total amount of structural components (NDF) increases in old pads. However, no significant increase in structural components was obtained after treatment (except for the OM.Acetic.C sample). This rather contradictory result may be due to the variability of the collected pads: the three selected OM plants were of different sizes and different localisations, and all samples were not collected on the same day, so the results can be influenced by abiotic factors and the age of the cladodes.

Comparing the results of the untreated samples (OM and OM.Old), it can be observed that cellulose and lignin content increases with the age of the plant, while hemicellulose content is more variable. It is somewhat surprising that the OM.Old.A sample shows significantly lower cellulose and lignin values than those obtained for OM.Old.B and OM.Old.C samples. A possible explanation for this might be that OM plant A was much smaller than plants B and C, so the number of cladodes that old pads had to support was lower, and therefore the structural components were also lower.

For OD cladodes, the NDF content increases in all treated samples, for example, up to 10% after water treatment (from 61.69% to 68.75%) and up to 33% after acid treatment (from 61.69% to 82.95%) for plant B. In this case, the three selected plants were quite similar to each other—they were from the same area and all the samples were collected the same day—so the results accord with the objective of the treatments, that is, to remove non-structural components. All components (cellulose, hemicellulose, and lignin) increase after treatment for the three OD plants. These data suggest that acetic-acid-treated OD cladodes could be evaluated as reinforcement for polymeric matrices as an alternative to the use of their fibre (due to the difficulty of obtaining it).

According to the results from Table 3, OM cladodes have a lower content of NDF (41.54–58.49%) and hemicellulose (25.15–42.60%) than OD cladodes (59.68–61.83% and 50%, respectively), whereas cellulose content is slightly higher (approximately 13% for OM versus 10% for OD) and lignin content is similar (1.63–3.72% for both species). However, considering the variability of the samples, these data must be interpreted with caution, because the findings might not be transferable.

If the structural components (NDF) are added with the non-structural components (ash and CP), values that do not reach 100% are obtained (these are between 65% and 80%). Based on the bibliography, it is considered that the main non-quantified component is mucilage: it can constitute 14–20% of the dry weight of the cladodes [34,50]. It consists of two fractions: one with gelling properties Ca^2+^ (pectin) and another without gelling properties [50].

#### 3.1.3. FTIR Analysis

FTIR spectroscopy is a useful tool for functional group identification on the cladode surface, allowing one to analyse if the treatments produce any surface modification on them. The FTIR spectra of both species of cladodes are shown in Figure 4. The peak positions and the possible assignments of functional groups for each sample are shown in Table A1 (Appendix A).

All samples present similar spectra, with any superficial modification by the treatments not being appreciable. The two bands around 3000 and 2800 cm^−1^ (peaks “b” and “c”) are related to asymmetric and symmetric methyl and methylene stretching groups present in the spectra of all of the fibre components, but most notably in the spectra for cellulose. The peak around 2850 cm^−1^ may also be due to a greater presence of hemicellulose and extractive [51].

The peak “d” (around 1715 cm^−1^) is more prominent in the OD spectrum. This might be attributed to the higher hemicellulose content in these samples compared with OM samples. These results are in agreement with the results of the composition (Table 3): OM samples show a hemicellulose content between 30% and 40%, whereas OD samples show between 50% and 60%.

The peak “f” (around 1515 cm^−1^) is assigned to aromatic C=O stretching in lignin, but it could also correspond to unsaturation (C=C) in the molecules, which may be related to high content in extractives and lignin [52,53]. It is observed in OM samples but not in OD samples. In contrast, the peak “g” (around 1400 cm^−1^) is more intensive in OD samples than in OM samples. This band is associated with the alkanes groups of hemicellulose and pectin [54], corroborating the chemical composition results (higher hemicellulose content in OD than OM samples). Table A1 from Appendix A shows more details of the peak position of the different samples and the possible assignments of functional groups.

### 3.2. Fibre Characterisation

**Figure 5:**
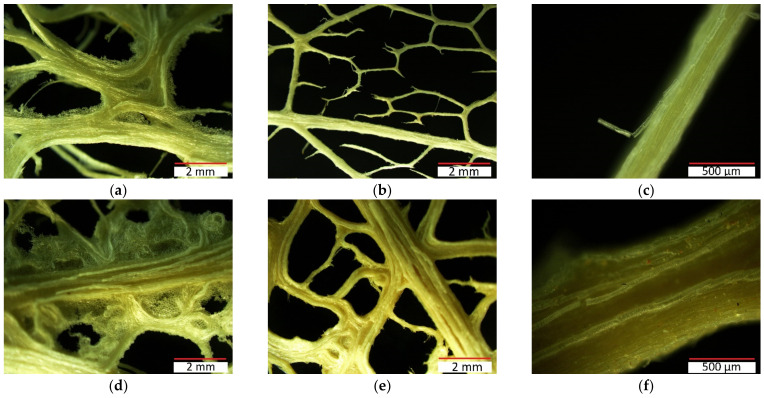
Optical microscope images of fibres bundles obtained by water retting process from OM young cladodes (**a**–**c**) and old cladodes (**d**–**f**).

**Figure 6:**
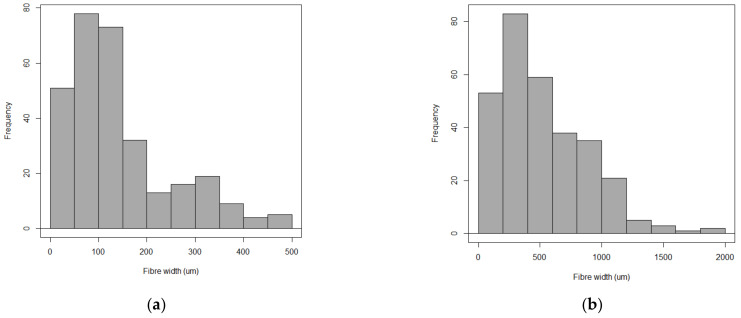
Histogram of fibre bundle width (µm) from: (**a**) young OM cladodes (n = 300), (**b**) old OM cladodes (n = 300).

**Figure 7:**
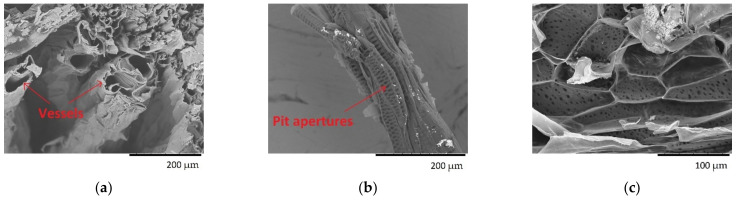
SEM images of *Opuntia* fibre bundles: (**a**) vessels, (**b**) tracheid with pit apertures, (**c**) periderm cell.

**Figure 8:**
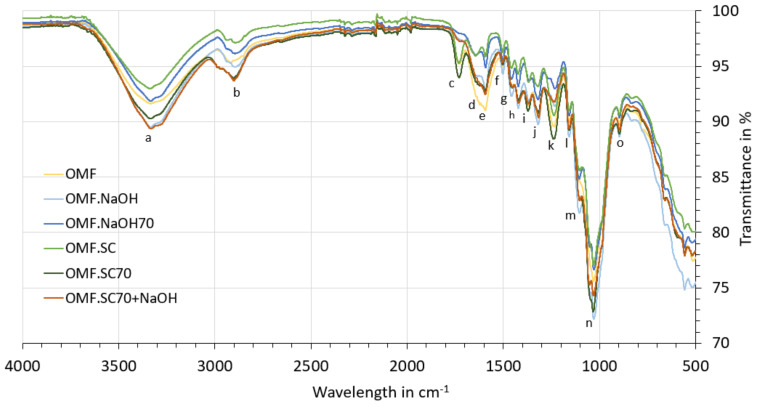
FTIR spectra for untreated and treated OM fibres.

**Figure 9:**
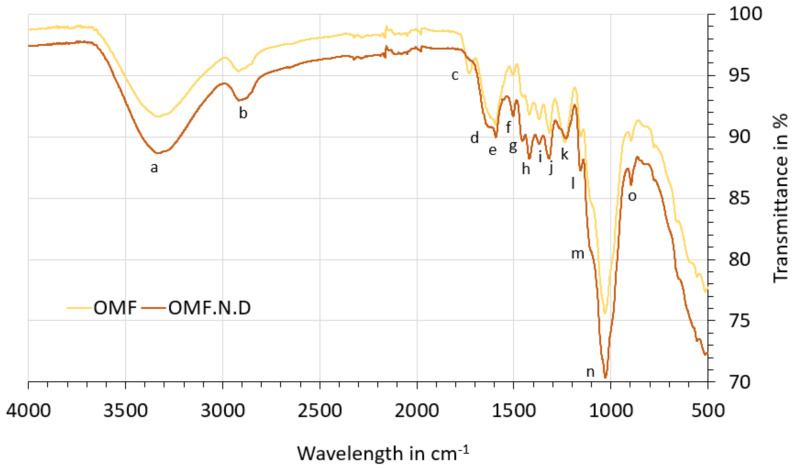
FTIR spectra (comparison between fibres directly collected and fibres extracted in water).

This section describes the data obtained for the fibre. Firstly, dimensions taken with the optical microscope (Figure 5 and Figure 6) and SEM images (Figure 7) are shown, then the fibre chemical composition is presented (Table 4 and Table 5), and finally the FTIR spectrum are provided (Figure 8 and Figure 9).

#### 3.2.1. Fibre Bundle Morphology

The hexagonal reticular hierarchical structure is considered as the main characteristic of *Opuntia* fibre [19]. Figure 5 shows different images of fibre network structure where it can be appreciated that it is similar to a honeycomb (Figure 5a,d). Moreover, it can be seen that the *Opuntia* skeleton is formed by fibres with a random orientation that create a network where two types of fibres can be distinguished: primary axial fibres (thicker) reticulated by secondary fibres (thinner) (Figure 5b,e), as reported by Mannai et al., (2018) [18]. This *Opuntia* fibre network is formed by the union of cellulose microfibres (Figure 5c,f).

Figure 6 corroborates the idea that the *Opuntia* network is formed by two types of fibres (primary and secondary). Fibre bundles obtained from young cladodes are thinner (50–150 µm on average) than fibre from old cladodes (200–400 µm on average), as can also be appreciated in Figure 5.

Vegetal fibres have a length between 8 and 14 mm and a diameter between 10 and 50 µm, approximately [55]. In the case of the *Opuntia* fibre network, they show different diameters: larger than 100 µm to vessels (Figure 7a) and between 12 and 30 µm to tracheids of the axial parenchyma cells (Figure 7b) [18]. Considering this and the width of the fibre bundles measured in this work, *Opuntia* fibre bundles can be formed by 4–10 cells in the case of the fibre obtained from young cladodes, and by more than 10 cells in the case of the fibre obtained from old cladodes.

#### 3.2.2. Chemical Composition

The results obtained from the preliminary analysis of fibre composition are shown in Table 4. It shows a comparison between the two *Opuntia* species under study as well as some first results of the effect of alkaline treatment. The fibre ash content is lower than the cladode ash content due to its cleaning with water, while the cellulose and lignin content increases in the fibre due to removal of hemicellulose and other polysaccharides. Cellulose content increases (up to 12% for OM and up to 17% for OD) after the fibre treatment. These results have important implications for developing composites because in general, the higher the cellulose content, the better the mechanical properties [56].

No differences were found between OM fibre and OD fibre.

**Figure A1:**
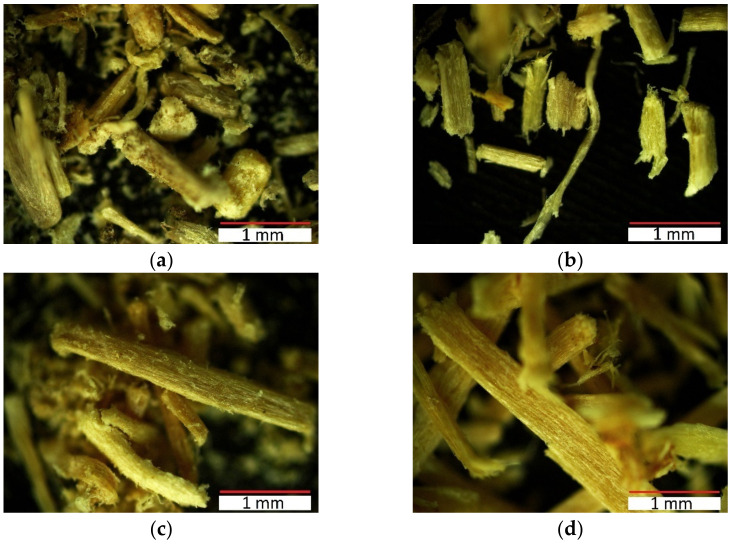
Optical microscope images of ground fibres obtained directly from nature: (**a**) OMF.N.D, (**b**) OMF.N.NaOH.D, (**c**) ODF.N.D, (**d**) ODF.N.NaOH.D.

In Figure A1 (Appendix A), it can be observed that the treated fibres show a smoother surface than the untreated fibre, possibly due to the partial removal of extractives, hemicellulose, and other elements.

Table 5 presents the composition results of treated and untreated fibres. A similar composition is observed for OMF.Young and OMF samples for plants B and C, but it does not happen in A. Regarding the treatment with NaOH, there are no differences between the treatment at room temperature and the treatment at 70 °C. In both cases the cellulose content increases (although in plant B it is not so appreciable), as does the lignin, while the hemicellulose content slightly decreases. Considering the treatment with sodium chlorite, the proportion of cellulose increases slightly both at room temperature and at 70 °C, whereas the lignin content only decreases when the fibre is treated at 70 °C. Combining the treatment with sodium chlorite and NaOH, it is possible to increase the cellulose content up to 24% and decrease the lignin content up to 86%. The better yields are achieved for SC-treated samples (independently of temperature), probably due to alkaline treatments removing more non-structural components.

#### 3.2.3. FTIR Analysis

The absorption bands for characteristic chemical groups of the lignocellulosic fibre can be observed in Figure 8. The peak positions and the possible assignments of functional groups for each sample are shown in Table A2 (Appendix A). The main difference is observed in peak “c” (around 1730 cm^−1^), which disappears in the OMF.NaOH, OMF.NaOH70, and OMF.SC70NaOH samples. This band is associated with the C=O carboxylic acid groups of pectin and hemicelluloses [54,57], so these findings suggest that NaOH removes part of them from the fibre surface. The peak “f” (around 1500 cm^−1^) reduces its height after SC70 treatments; this band is attributed to aromatic C=O stretching in lignin [58], so its reduction is in agreement with the compositions results. The peak “k” (around 1230 cm^−1^), associated with C-O stretching in the acetyl groups in hemicelluloses, decreases its intensity in the OMF.NaOH, OMF.NaOH70, and OMF.SC70NaOH samples. The intensity of the peak “m” (around 1104 cm^−1^) increases for all treated samples; it is attributed mainly to C-O-C glycosidic ether from cellulose [53]. This increase in cellulose exposed on the surface increases the number of reaction sites, and consequently, a strong adhesion with the polymeric matrix can be achieved.

If the spectra of fibres (Figure 8) are compared with those from cladodes (Figure 4), it is observed that in the fibre samples, the peaks around 2850 cm^−1^ and 777 cm^−1^ disappear. This reflects a lower presence of extractives and hemicellulose in the fibre surface than in cladodes [51,52,59], and also a lower ash content. The band around 777 cm^−1^ has been attributed to OCO deformations of calcium oxalate presents in the *Opuntia* cladodes [60].

Finally, it is interesting to compare the OMF spectrum and OMF.N spectrum (Figure 9). The peak “c” around 1730 cm^−1^ is only observed for OMF samples. Considering the compositions results, where there is not a significant difference between OMF (57–65% cellulose, 8–13% hemicellulose, and 6–10% lignin) and OMF.N (55% cellulose, 13% hemicellulose, 11% lignin) samples, so it seems possible that this difference is due to the presence of pectin in the fibre obtained by the water retting process. Fibres obtained by water retting process have a more jelly-like appearance than fibres directly collected from nature (Figure 2). The intensity of the peaks “f” and “h” are higher for the OMF.N sample, which can be attributed to a higher lignin content due to the maturity of the fibre. There is a reduction in the peak height at peak “k” for OMF.N, consistent with a lower presence of hemicelluloses and pectins in these samples.

## 4. Discussion

Biometric parameters obtained are similar to those reported in previous studies (34.9 cm length and 17.6 cm width) [47].

Results obtained for the chemical composition of *Opuntia* cladodes are between the values found in the literature: a variable protein content (3–18%), a high ash content (15–33%), a low lignin content (1–4%), and similar values of cellulose (6–27%) and hemicellulose (15–27%) [17,35,36,61,62]. Higher levels of hemicellulose have been obtained in this study, reaching up to 50%. This difference might be due to abiotic factors (soil, climate, etc.), characterisation methods used, post-harvest handling, vegetative state, or plant age, among others factors [18,34,38,39,40].

According to other studies, OM cladodes have a higher ash and structural components content and a lower moisture content than OD cladodes [47]. In this study, these results depend more on the plant than on the species analysed. For this reason, the comparative results between both species are not considered generalisable.

Considering the cladodes treatments, acetic acid was used because acidic medium can facilitate the *Opuntia* fibres separation, obtaining a uniform structure and removing part of the lignin which covers the fibres’ outer surface [36]. In this study, it was not possible to remove lignin from the surface (FTIR spectra do not reflect modifications), and an increase in all structural components is only observed in OD cladodes after the acid treatment. It is possible that acid facilitates the removal of pectins from the pads.

There is a difference between the composition of young and old cladodes, showing for the latter a higher content in structural components, in line with the results reported by Ventura-Aguilar et al. (2017) [34]. However, the chemical composition and FTIR analysis results do not reveal differences between the fibre extracted by water retting process from young and old cladodes, although morphologically, they show different geometric parameters. Fibre from old cladodes is thicker, and according to the previous studies, it can be assumed that it presents better mechanical properties due to a higher fibre density and a smaller area of the mesh pores [18,19].

In general, fibres with a higher cellulose content have better mechanical properties [63], because cellulose provides strength, stiffness, and dimensional stability to the fibre [64]. In accordance with this study, *Opuntia* fibre has a cellulose content between 50% and 67%. This cellulose content is greater than bamboo (26–43%) and coir (32–43%) fibres, and is closer to those of kenaf (31–72%) and abaca (56–63%) fibres [6]. Lignin can protect fibre from thermal and biological degradation [65]. In this study, *Opuntia* fibre show a lignin content between 6% and 13%. Only one study has been found that studied the *Opuntia* fibre’s composition; it reported a lower lignin content (4.8%) and similar hemicellulose (10.9%) and cellulose (53.6%) contents [48]. Therefore, these results can promote the use of *Opuntia* fibre as reinforcement for polymeric matrices.

The alkaline fibre treatment under optimal conditions can improve its mechanical properties as a reinforcement for polymer matrices, increasing the tensile and flexural strength [66,67]. Analysing the fibre treatment effect, the modifications made by the alkaline treatment are clearly observed in the FTIR spectra, and they are of greater importance than those obtained with the sodium chlorite treatment. Hemicelluloses and pectins are removed from the fibre surface after NaOH treatment, increasing the cellulose content. These findings are consistent with other studies: pineapple crown fibres increased their cellulose content from 17.4% to 53.3% [68] after alkaline treatment, and *Agave americana* fibres from 68.54% to 78.65% [69]. Alkaline treatment can also reduce the lignin content from vegetal fibres [70,71], but the compositional results indicate an increase in lignin (at room temperature and at 70 °C). It seems possible that these findings are due to the short treatment period and the method used to determine the structural components. A lignin reduction is only achieved with the SC treatment at 70 °C.

Kenaf fibres have been treated under similar conditions, increasing the cellulose content from 56.81% to 65.24% after the alkaline treatment and decreasing the hemicellulose and lignin content from 13.59% to 10.42% and from 18.27% to 15.93%, respectively. A greater increase in the cellulose content is achieved (from 56.81% to 75.92%) by combining the alkaline treatment with an SC treatment, due to a greater removal of hemicellulose (from 13.59% to 9.03%) and lignin (from 18.27% to 8.03%). The alkaline treatment also improves the thermal stability of the fibres, whereas the alkaline and SC treatments together have a greater effect on increasing the tensile strength than the alkaline treatment alone [58]. The alkaline treatment of jute fibres increased the tensile strength of a natural rubber from 10.52 MPa (with untreated jute fibres) to 14.21 MPa (with treated jute fibres) [72]. On the other hand, bleaching treatments with SC increased the tensile strength of sisal fibres and polyester composites reinforced with it [71], and poly(methyl) methacrylate reinforced with birch veneer [44]. Therefore, further work is required to establish if it is better to use an alkaline treatment or a combination of alkaline treatment with SC.

Finally, no references have been found comparing *Opuntia* fibre directly obtained from nature and *Opuntia* fibre obtained by water retting process. The first method reduces the time needed to obtain the raw material (as natural processes happening in the plant itself are degrading the non-fibrous part of the pad). On the other hand, the use of the water retting process, even if taking some weeks to allow fibre extraction, leads to more homogeneous fibres, with higher cellulose content and with more controlled properties. Further research might explore what conditions are better to obtain the fibre and use it as reinforcement in terms of economic and environmental viability.

The chemical nature of fibre as reinforcement has an important influence on the properties of the polymeric composites [73]. Therefore, the results obtained in this study are expected to enhance the knowledge about the composition of untreated and treated *Opuntia* fibre in order to exploit it as a reinforcement for polymeric matrices. The untreated and treated samples will be combined with different polymeric matrices using different process such as injection (short fibres bundles) or compression (short fibres bundles or fibre network). Considering the composition results, it is expected that the treatment with NaOH or SC70 can improve the fibre–matrix compatibility and consequently the mechanical properties of the polymeric matrices, showing better results than those reported up to now.

## 5. Conclusions

Despite the variability of wild plant materials, this paper has investigated the composition of *Opuntia* cladodes and fibre. It can be considered one of the few studies focusing on *Opuntia* fibre composition to date. Fibre structural components results (50–66% cellulose, 8–13% hemicellulose, and 6-14% lignin) suggest that it can be used for obtaining composites.

*Opuntia* fibre can be extracted from OM cladodes without the need for chemicals or a laborious process, and it can also be collected directly from nature. In this study, their chemical composition has been compared, although future research to compare their mechanical properties as reinforcement is needed.

Obtaining the fibre from OM is easier than from OD. However, OD cladodes can be evaluated as reinforcement. Considering that vegetal fibres’ mechanical properties depend on their chemical composition, OD cladodes properties can be improved by acetic acid treatment, which allows one to remove non-structural components.

Chemical composition and FTIR results of untreated and treated fibre suggest that NaOH treatment at room temperature can improve the fibre–matrix compatibility due to the removal of hemicelluloses and pectin from the fibre surface. Moreover, sodium chlorite treatment at 70 °C reduces the lignin content.

Future research will focus on analysing the effect of the treatments on the thermal stability of the fibres and obtaining composites that combine untreated and treated *Opuntia* fibres and polymer matrices. Mechanical properties of these composites will be analysed in order to assess if the treatments have a positive influence on the fibre properties as reinforcement, which is expected considering the composition results.

## Figures and Tables

**Table 1:** Summary and identification of all *Opuntia* samples obtained.

Specie	Plant	Part	Treatment	Code
*O. maxima*	A, B, C	Young cladode	-	OM
			Water	OM.H_2_O
			Acetic acid	OM.Acetic
		Old cladode	-	OM.Old
		Fibre from young cladode (extracted in water)	-	OMF.Young
		Fibre from old cladode (extracted in water)	-	OMF
			NaOH	OMF.NaOH
			NaOH 70 °C	OMF.NaOH70
			Sodium chlorite	OMF.SC
			Sodium chlorite 70 °C	OMF.SC70
			Sodium chlorite 70 °C + NaOH	OMF.SC70 + NaOH
	D	Fibre directly collected from nature	-	OMF.N
			NaOH	OMF.N.NaOH
*O. dillenii*	A, B, C	Young cladode	-	OD
			Water	OD.H_2_O
			Acetic acid	OD.Acetic
	D	Fibre directly collected from nature	-	ODF.N
			NaOH	ODF.N.NaOH

(-) this symbol indicates untreated samples.

**Table 2:** Biometric parameters measured in the *O. maxima* cladodes.

Sample	Length in cm	Width in cm
OM.A	33.9 ± 4.8	13.6 ± 1.2
OM.B	35.3 ± 2.9	17.7 ± 1.3
OM.C	36.2 ± 4.2	18.5 ± 2.8

Results show the mean value ± standard deviation of 10 replicates.

**Table 3:** Chemical composition of *Opuntia* cladodes (% wt in dry base).

Scheme	Yield	Moisture	Ash	CP	NDF	Cellulose	Hemicellulose	Lignin
OM.A	-	9.37 ± 0.01	20.55 ± 0.22	2.67 ± 0.12	51.91 ± 2.01	13.60 ± 0.31	36.57 ± 1.86	1.74 ± 0.08
OM.H_2_O.A	7.8	7.32 ± 0.04	23.82 ± 0.07	2.62 ± 0.04	52.53 ± 2.56	12.81 ± 0.62	37.88 ± 2.09	1.85 ± 0.06
OM.Acetic.A	5.8	5.45 ± 0.02	20.01 ± 0.03	3.88 ± 0.02	42.09 ± 2.97	12.88 ± 0.54	27.46 ± 2.36	1.75 ± 0.08
OM.Old.A	-	24.01 ± 0.04	15.41 ± 0.31	1.59 ± 0.17	56.41 ± 2.22	17.78 ± 0.08	36.35 ± 1.85	2.27 ± 0.39
OM.B	-	7.41 ± 0.04	19.53 ± 0.25	2.71 ± 0.03	58.49 ± 2.20	13.86 ± 0.20	42.60 ± 2.24	2.03 ± 0.18
OM.H_2_O.B	7.2	6.45 ± 0.01	19.46 ± 0.02	3.52 ± 0.15	40.22 ± 1.71	10.62 ± 0.42	26.90 ± 1.44	2.70 ± 0.16
OM.Acetic.B	4.6	4.26 ± 0.01	22.17 ± 0.14	5.04 ± 0.54	43.22 ± 5.87	12.73 ± 0.75	27.66 ± 4.98	2.83 ± 0.18
OM.Old.B	-	10.03 ± 0.00	12.58 ± 0.07	2.06 ± 0.10	69.37 ± 1.37	26.09 ± 0.42	31.19 ± 1.00	12.09 ± 0.78
OM.C	-	5.40 ± 0.02	18.75 ± 0.24	3.82 ± 0.42	41.54 ± 4.12	12.68 ± 0.38	25.15 ± 3.83	3.72 ± 0.62
OM.H_2_O.C	5.9	5.12 ± 0.01	19.53 ± 0.21	6.41 ± 0.29	36.07 ± 2.38	11.58 ± 0.19	21.56 ± 2.39	2.93 ± 0.18
OM.Acetic.C	3.5	3.14 ± 0.00	10.41 ± 0.33	7.69 ± 0.33	54.24 ± 3.96	16.11 ± 0.11	34.23 ± 23.84	3.89 ± 0.03
OM.Old.C	-	15.58 ± 0.04	5.43 ± 0.13	1.60 ± 0.16	72.82 ± 1.38	32.68 ± 1.03	30.21 ± 1.14	9.93 ± 0.81
OD.A	-	8.51 ± 0.31	19.00 ± 0.10	2.78 ± 0.04	59.68 ± 0.85	9.07 ± 0.53	49.53 ± 1.18	1.63 ± 0.18
OD.H_2_O.A	9.6	8.53 ± 0.02	16.37 ± 0.25	2.79 ± 0.10	63.38 ± 0.52	11.20 ± 0.63	50.27 ± 0.94	1.91 ± 0.17
OD.Acetic.A	5.1	4.61 ± 0.01	11.66 ± 0.58	3.63 ± 0.07	78.20 ± 0.38	16.03 ± 0.76	59.36 ± 0.45	2.82 ± 0.26
OD.B	-	7.54 ± 0.32	18.19 ± 0.10	3.55 ± 0.26	61.69 ± 0.98	10.48 ± 0.20	49.48 ± 1.26	1.73 ± 0.16
OD.H_2_O.B	9.5	8.34 ± 0.45	17.36 ± 0.22	3.31 ± 0.05	68.75 ± 1.79	12.60 ± 0.47	53.93 ± 2.11	2.22 ± 0.26
OD.Acetic.B	4.5	4.10 ± 0.01	14.23 ± 0.17	3.87 ± 0.09	82.05 ± 4.83	17.68 ± 1.06	57.40 ± 0.40	3.35 ± 0.55
OD.C	-	8.61 ± 0.04	18.28 ± 0.37	2.60 ± 0.03	61.83 ± 0.47	9.86 ± 0.37	50.20 ± 0.28	1.77 ± 0.12
OD.H_2_O.C	9.8	8.38 ± 0.17	19.53 ± 0.29	3.44 ± 0.21	63.48 ± 1.67	14.00 ± 0.72	47.07 ± 1.28	2.42 ± 0.14
OD.Acetic.C	4.5	4.28 ± 0.24	15.20 ± 0.14	4.69 ± 0.02	78.65 ± 0.77	18.57 ± 1.24	56.77 ± 1.24	3.31 ± 0.09

Results show the mean value ± standard deviation of 3 replicates.

**Table 4:** Composition of *O. maxima* and *O. dillenii* fibre (% wt in dry base).

Sample	Moisture	Ash	CP	Cellulose	Hemicellulose	Lignin
OMF.N.D	6.20 ± 0.25	3.45 ± 0.22	1.97 ± 0.09	55.37 ± 0.91	13.07 ± 0.69	11.36 ± 0.22
OMF.N.NaOH.D	5.83 ± 0.53	1.12 ± 0.15	-	62.25 ± 0.34	9.51 ± 0.27	10.22 ± 0.37
ODF.N.D	5.51 ± 0.15	6.67 ± 0.70	1.33 ± 0.07	50.32 ± 0.62	11.57 ± 0.56	14.11 ± 0.51
ODF.N.NaOH.D	5.23 ± 0.48	1.49 ± 0.13	-	58.91 ± 1.01	9.72 ± 0.79	13.52 ± 0.58

Results show the mean value ± standard deviation of 3 replicates.

**Table 5:** Composition of *Opuntia* fibre (% wt in dry base).

Sample	Yield	Moisture	Ash	NDF	Cellulose	Hemicellulose	Lignin
OMF.Young.A	-	6.14 ± 0.50	1.46 ± 0.30	81.78 ± 0.23	66.88 ± 0.31	8.64 ± 0.29	6.26 ± 0.32
OMF.A	-	8.12 ± 1.01	0.93 ± 0.22	78.80 ± 1.00	56.88 ± 0.15	13.69 ± 0.87	8.23 ± 0.35
OMF.NaOH.A	64.4	8.33 ± 0.17	0.57 ± 0.11	84.31 ± 0.49	63.07 ± 0.77	11.47 ± 0.76	9.77 ± 0.58
OMF.NaOH70.A	65.6	5.52 ± 0.46	0.84 ± 0.31	87.07 ± 0.10	65.40 ± 0.50	11.51 ± 0.42	10.17 ± 0.46
OMF.SC.A	71.6	5.83 ± 0.67	0.37 ± 0.18	86.1 ± 0.32	62.52 ± 0.66	14.74 ± 0.59	8.85 ± 0.86
OMF.SC70.A	77.4	3.58 ± 0.67	0.96 ± 0.04	86.07 ± 0.54	65.62 ± 1.91	17.88 ± 0.21	3.85 ± 0.20
OMF.SC70NaOH.A	69.3	4.68 ± 0.94	0.78 ± 0.24	86.92 ± 0.17	70.38 ± 0.33	12.68 ± 0.31	3.78 ± 0.29
OMF.Young.B	-	7.40 ± 0.33	0.34 ± 0.17	81.04 ± 0.33	64.00 ± 0.22	9.81 ± 0.22	7.23 ± 0.18
OMF.B	-	7.16 ± 0.47	0.77 ± 0.17	80.89 ± 0.71	65.07 ± 1.01	8.44 ± 0.32	7.37 ± 0.17
OMF.NaOH.B	68.4	6.34 ± 0.27	0.74 ± 0.08	85.35 ± 0.37	66.97 ± 0.64	8.11 ± 0.74	10.27 ± 0.26
OMF.NaOH70.B	65.6	7.35 ± 1.52	1.12 ± 0.37	84.74 ± 0.31	66.11 ± 0.28	7.84 ± 0.59	10.79 ± 0.45
OMF.SC.B	79.6	5.95 ± 1.01	0.68 ± 0.02	86.73 ± 0.45	69.24 ± 0.24	10.00 ± 0.80	7.49 ± 0.71
OMF.SC70.B	78.2	6.87 ± 0.27	1.00 ± 0.22	81.32 ± 0.08	68.46 ± 0.47	11.91 ± 0.82	0.95 ± 0.29
OMF.SC70NaOH.B	63.6	7.03 ± 0.25	1.69 ± 0.47	83.43 ± 0.24	74.20 ± 0.32	8.23 ± 0.49	1.00 ± 0.17
OMF.Young.C	-	6.57 ± 0.04	0.77 ± 0.36	79.58 ± 1.70	62.81 ± 1.63	10.93 ± 0.39	5.85 ± 0.19
OMF.C	-	6.25 ± 0.39	0.57 ± 0.24	79.85 ± 0.39	62.63 ± 1.87	7.71 ± 0.38	9.52 ± 1.26
OMF.NaOH.C	66.0	7.78 ± 0.19	0.83 ± 0.51	84.85 ± 0.19	65.92 ± 0.73	6.45 ± 0.43	12.48 ± 0.58
OMF.NaOH70.C	64.8	8.08 ± 0.14	0.95 ± 0.36	84.87 ± 0.13	64.88 ± 0.67	6.16 ± 0.68	13.83 ± 0.14
OMF.SC.C	82.4	8.38 ± 1.39	0.53 ± 0.13	80.93 ± 0.11	65.24 ± 0.86	6.93 ± 0.06	8.75 ± 0.78
OMF.SC70.C	82.0	6.90 ± 0.50	0.73 ± 0.11	81.15 ± 0.03	68.85 ± 0.39	7.77 ± 0.16	4.54 ± 0.47
OMF.SC70NaOH.C	73.3	3.68 ± 0.63	1.25 ± 0.13	83.75 ± 0.08	67.81 ± 0.70	9.91 ± 0.51	6.03 ± 0.33

Results show the mean value ± standard deviation of 3 replicates.

## Data Availability

Not applicable.

## Appendix A

**Table A1:** FTIR spectral data for treated and untreated OM and OD cladodes.

Peak Label	Wavelength	Peak Position in cm^−1^							Possible Assignment [58,74,75]
		OM	OM.H_2_O	OM.Acetic	OM.Old	OD	OD.H_2_O	OD.Acetic	
a	3400–3000	3271 ± 0.1	3271 ± 0.2	3269 ± 0.3	3327 ± 7.0	3281 ± 0.4	3280 ± 0.5	3298 ± 18	O–H stretching vibration
b	3000–2900	2920 ± 0.8	2919 ± 0.3	2919 ± 0.4	2919 ± 2.0	2918 ± 0.2	2918 ± 0.3	2918 ± 0.4	C–H stretching vibration
c	2900–2800	2850 ± 0.6	2851 ± 0.3	2850 ± 0.2	2850 ± 0.3	2850 ± 0.1	2850 ± 0.3	2851 ± 0.4	C–H stretching vibration
d	1750–1690	1717 ± 5.5	1717 ± 2.2	1714 ± 4.6	1712 ± 2.2	1713 ± 0.1	1715 ± 3.7	1715 ± 3.1	=CO stretching vibration of carboxylic acid and ester groups of hemicellulose
e	1690–1600	1614 ± 0.1	1614 ± 0.1	1611 ± 0.3	1612 ± 4.3	1592 ± 0.8	1595 ± 5.2	1614 ± 0.1	Aromatic C=O stretching in lignin
f	1550–1500	1515 ± 0.2	1515 ± 0.1	1515 ± 0.3	1515 ± 0.4	*	*	*	Aromatic C=O stretching in lignin
g	1500–1390	1417 ± 0.01	1417 ± 0.1	1420 ± 1.2	1416 ± 1.0	1393 ± 0.1	1393 ± 0.4	1393 ± 0.2	*CH*_2_ bending
h	1390–1360	1372 ± 0.2	1372 ± 0.1	1371 ± 0.6	1371 ± 1.5	*	*	1372 ± 0.1	*C*–*H* bending
i	1350–1300	1315 ± 0.2	1315 ± 0.1	1316 ± 0.3	1318 ± 1.1	1316 ± 0.3	1315 ± 0.3	1315 ± 0.2	Aromatic C–O bending
j	1300–1200	1243 ± 0.3	1243 ± 0.1	1243 ± 0.3	1242 ± 0.2	1249 ± 0.6	1249 ± 0.6	1241 ± 0.1	–CO stretching of acetyl group
k	1200–1100	1143 ± 0.1	1141 ± 2.2	1141 ± 1.6	1146 ± 1.0	1144 ± 1.5	1147 ± 2.8	1146 ± 1.0	Antisymmetric deformation of C–O–C band
l	1100–910	1033 ± 2.0	1035 ± 0.1	1031 ± 0.5	1034 ± 5.3	1037 ± 0.2	1037 ± 0.8	1037 ± 0.1	C–O stretching vibration in cellulose
m	910–840	892 ± 0.5	892 ± 0.3	892 ± 0.6	893 ± 0.6	892 ± 0.5	892 ± 0.3	893 ± 0.5	β–Glucosidic linkage in cellulose
n	840–650	777 ± 0.1	777 ± 0.1	778 ± 0.3	776 ± 2.0	778 ± 0.1	778 ± 0.4	779 ± 0.5	C–OH out-of-plane bending

* Indicates the absence or decrease of the peak. Results show the mean value ± standard deviation of 3 replicates.

**Table A2:** FTIR spectral data for treated and untreated *OM* fibres.

Peak Label	Wavelength	Peak Position in cm^−1^						Possible Assignment [58,74,75]
		OMF	NaOH	NaOH70	SC	SC70	SC70NaOH	
a	3400–3000	3333 ± 0.5	3334 ± 0.9	3334 ± 0.3	3336 ± 1.2	3335 ± 0.8	3333 ± 0.5	O–H stretching vibration
b	3000–2900	2919 ± 0.4	2896 ± 0.7	2894 ± 1.6	2889 ± 9.5	2900 ± 0.3	2901 ± 0.1	C–H stretching vibration
c	1750–1690	1728 ± 0.1	*	*	1731 ± 1.0	1729 ± 0.6	*	=CO stretching vibration of carboxylic acid
d	1690–1600	*	1642 ± 0.2	1645 ± 0.9	1645 ± 1.3	1627 ± 0.5	*	Absorbed water
e	1600–1550	1594 ± 0.1	1592 ± 0.4	1592 ± 0.3	1593 ± 0.5	1594 ± 0.3	1593 ± 0.4	Aromatic C=O stretching in lignin
f	1550–1500	1502 ± 0.4	1503 ± 0.4	1503 ± 0.6	1504 ± 1.0	1503 ± 0.6	1504 ± 1.4	Aromatic C=O stretching in lignin
g	1500–1450	1452 ± 0.8	1458 ± 0.7	1457 ± 2.1	1457 ± 2.1	1454 ± 2.5	1453 ± 1.3	*CH*_2_ bending
h	1450–1390	1421 ± 0.9	1422 ± 0.1	1422 ± 0.3	1422 ± 0.6	1423 ± 1.5	1422 ± 0.4	*CH*_2_ bending
i	1390–1350	1370 ± 0.3	1368 ± 1.0	1367 ± 0.5	1369 ± 2.1	1371 ± 1.0	1371 ± 0.5	C-H bending
j	1350–1300	1318 ± 0.6	1318 ± 0.3	1319 ± 0.2	1320 ± 1.5	1317 ± 0.4	1316 ± 0.5	Aromatic C-O bending
k	1300–1200	1237 ± 0.6	1232 ± 1.9	1232 ± 0.6	1236 ± 0.5	1239 ± 3.1	1236 ± 4.4	–CO stretching of acetyl group
l	1200–1150	1156 ± 0.5	1158 ± 0.1	1158 ± 0.1	1158 ± 0.3	1158 ± 0.1	1159 ± 0.1	Antisymmetric deformation of C–O–C
m	1150–1100	*	1104 ± 1.3	1105 ± 0.7	1104 ± 0.7	1103 ± 0.7	1103 ± 0.4	C–C, C–OH, C–H ringand side group vibrations
n	1100–910	1031 ± 0.6	1030 ± 0.1	1030 ± 0.1	1031 ± 0.4	1032 ± 0.3	1030 ± 0.8	C–O stretching vibration in cellulose
o	910–840	896 ± 0.2	896 ± 0.1	896 ± 0.3	897 ± 0.7	897 ± 0.3	896 ± 0.1	β-Glucosidic linkage in cellulose

* Indicates the absence or decrease of the peak. Results show the mean value ± standard deviation of 3 replicates.
